# Ten circumstances and solutions for finding the sample mean and standard deviation for meta-analysis

**DOI:** 10.1186/s13643-023-02217-1

**Published:** 2023-04-01

**Authors:** Kuan-Yu Chi, Man-Yun Li, Chiehfeng Chen, Enoch Kang, Cochrane Taiwan

**Affiliations:** 1 School of Medicine, College of Medicine, Taipei Medical University, Taipei, Taiwan; 2 Department of Public Health, School of Medicine, College of Medicine, Taipei Medical University, Taipei, Taiwan; 3 Division of Plastic Surgery, Department of Surgery, Wan Fang Hospital, Taipei Medical University, Taipei, Taiwan; 4 Cochrane Taiwan, Taipei Medical University, 250 Wu-Hsing Street, Taipei, 110 Taiwan; 5 Evidence-Based Medicine Center, Wan Fang Hospital, Taipei Medical University, Taipei, Taiwan; 6 Research center of big data and meta-analysis, Wan Fang Hospital, Taipei Medical University, Taipei, Taiwan; 7 Institute of Health Policy and Management, College of Public Health, National Taiwan University, Taipei, Taiwan

**Keywords:** Data conversion, Data estimation, *p *value, *t *value, *z *score, Interquartile, Range

## Abstract

A common problem in meta-analyses is the unavailability of mean and standard deviation (SD). Unfortunately, only having values of the median, interquartile range (IQR), or range cannot be directly utilized for meta-analysis. Although some estimation and conversion methods have been proposed in the past two decades, there were no published and user-friendly tools developed based on multiple scenarios of missing SD. Therefore, this study aimed to provide a collection of possible circumstances of missing sample means or SD with solutions for teaching and research. A total of 10 common circumstances of missing SD or mean could have available statistics of *p* value, *t* value, *z *score, confidence interval, standard error, median, IQR, and range. Teachers and investigators can use relevant formulas for finding the sample mean and SD according to the available circumstance. Due to the complicated computations, our team provides a free available spreadsheet. With ever-evolving statistical methods, some formulas may be further improved in the future; therefore, it is recommended to involve statisticians in evidence-based practice or systematic reviews.

**Supplementary Information:**

The online version contains supplementary material available at 10.1186/s13643-023-02217-1.

## Background

Meta-analysis is a quantitative analytic method only to be applied within an appropriate context in a systematic review and is an important method for having an overview of the evidence body of a specific topic, but “meta-analysis metastasis” have raised concerns in the academic field [1, 2]. Problematic data processing or analysis hides in many syntheses that threaten the quality of the evidence [3]. A recent study demonstrated the underlying causes of the retraction of meta-analysis shifted from academic ethical violations to methodological flaws including inappropriate data conversion or estimation [4]. The retractions of meta-analysis manuscripts for violating the academic ethics such as conflicts of author sequences, plagiarism, or other issues by the Committee on Publication Ethics (COPE) have been decreased from 80% to 40% before 2020. On the other hand, methodological flaw becomes a critical reason for the retraction of meta-analysis manuscript with an increasing trend from < 10 to 40%. In this aspect, knowledge and ability of data conversion and estimation should be the core skills for meta-analysis researchers. The objective of this study was to raise discussion on the circumstances and current solutions without adequate data for meta-analysis. Accordingly, this article includes a collection of possible circumstances of missing sample means or standard deviation (SD) with solutions for teaching and research.

## Methods

At Cochrane Taiwan and Wan Fang Hospital, two senior researchers (C-F.C. and E.K.) reviewed the designated textbooks and systematic reviews and screened reference lists for other potential references. Additional systematic reviews were identified by the systematic search, and handbooks were broadly used in evidence-based medicine (Supplementary 1 and 2). Based on the identified references, they listed possible circumstances of no adequate data for meta-analysis and sought formulas for identifying sample mean or SD in each circumstance. Following double-checking the circumstances and solutions, the working group categorized the identified circumstances and solutions and built an Excel tool for estimation and conversion of each circumstance in which solutions are based on the Cochrane Handbook and the study by Wan et al. (Supplementary 3) [5–7].

## Results

**Fig. 1:**
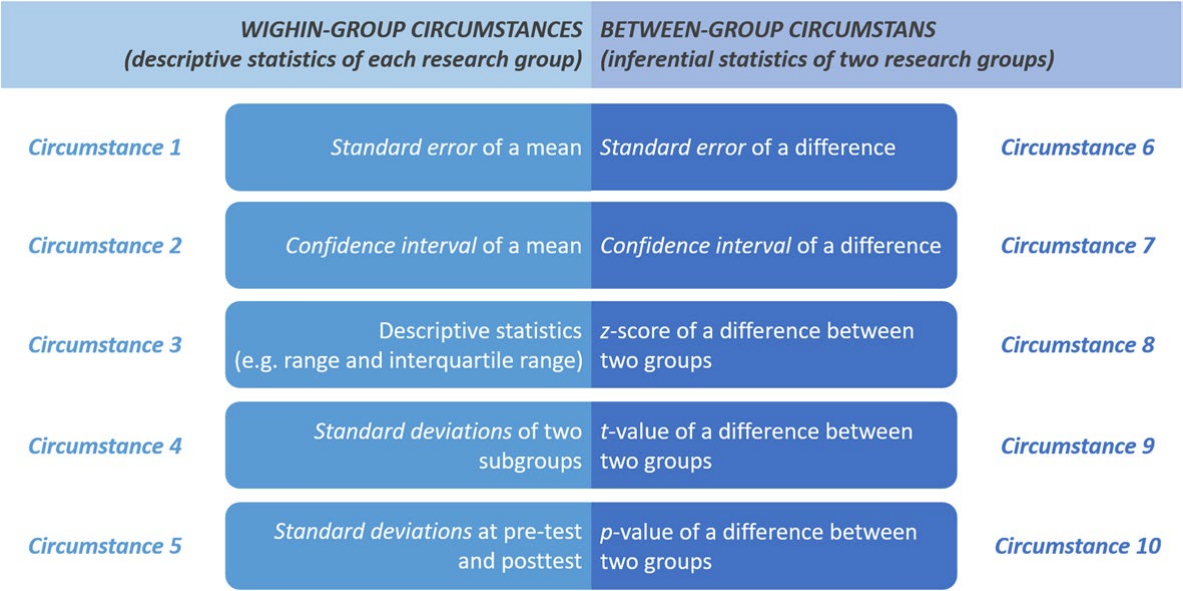
Ten convertible or estimable circumstances of the missing sample mean or standard deviation

The working group listed 10 principal circumstances and categorized them into two general categories, including (a) descriptive statistics of a single group (within-group circumstances) and (b) effect estimates of two treatment groups (between-group circumstances; Fig. 1).

### Within-group circumstances

#### Circumstance-1: only within-group standard error is available

Within-group SD can be obtained by multiplying within-group standard error (SE) by the square root of the sample size (formula-1; all formulas are placed in Supplementary 4) [6].

#### Circumstance-2: only within-group confidence interval is available

The confidence interval (CI) for a mean of a group can be converted to the corresponding SE and SD. If an original study has indicated what statistical test was used, investigators have to estimate SE and SD using formula-2 to formula-5 based on the corresponding distribution. According to the Cochrane Handbook, if a primary report does not describe which statistical test has been used, the sample size of each group (≧100 or < 100) might be a clue of the formula selection in terms of the decision between *z* distribution (formula 2 and formula 3) and *t* distribution (formula 4 and formula 5) [6].

#### Circumstance-3: descriptive statistics are available but no mean and SD

Investigators might estimate within-group mean and SD according to formulas in the Cochrane Handbook and an article by Hozo et al. [6, 8] when encountering the circumstance of no mean or SD but other descriptive statistics, including median, minimum, maximum, the first quartile, and the third quartile are available. For instance, SD could be simply estimated by dividing the interquartile range (IQR) by 1.35 [6], or selecting a formula according to the range of the sample size (formulas 6 and 7) [8]. However, the divisor of 1.35 only applies when the sample size is large and investigators may overestimate the SD when the sample size is small, which hinders general use. Although the sample size is taken into account in the selection of the conversion formula proposed by Hozo et al. [8], the formulas themselves are sample size-independent. Therefore, it would be more appropriate to use sample size-dependent formulas, which were modified and developed by Wan et al. [9] and Lou et al. [10] on the basis of Hozo’s [8] method. Because primary study may present descriptive statistics in different ways, investigators could find sub-circumstance with corresponding estimating formulas as follows:

##### Sub-circumstance 3.1: only minimum, median, and maximum are available

Lou et al. and Wan et al. proposed sample size-dependent methods for the estimation of mean and SD. Lou et al. introduced an optimal weight (*w*) into the approximation of a mean with a function of sample size (*n*) (formula 8) [7, 10]. Then, the mean could be estimated using the function of n with minimum, median, and maximum (formula 9). SD could be estimated by the function of *n* and range with the inverse function of the percentile of the standard normal distribution (formula 10).

##### Sub-circumstance 3.2: only median and quartiles are available

Similarly, modified methods result in more precise estimations for mean and SD from median and quartiles using formulas 11 and 12 [7, 8, 10, 11].

##### Sub-circumstance 3.3: minimum, Q1, median, Q3, and maximum are available

Estimations SD in this sub-circumstance, one can use the modification of Bland’s methods proposed by Lou et al. based on the same rationale (formulas 13 and 14) [10, 11].

##### Sub-circumstance 3.4: only range is available but without minimum and maximum

The formulas regarding estimations of the mean and SD from minimum and maximum can work if the original report provides a range without minimum and maximum [7, 10]. In consequence, sub-circumstance 3.4. is based on the formula 10 in sub-circumstance 3.1. Nevertheless, formula 9 cannot estimate a mean from range without minimum and maximum, the median is regarded as the mean in sub-circumstance 3.4 if data do not violate the assumption of normality.

##### Sub-circumstance 3.5: only interquartile range is available but without Q1 and Q3

The formulas regarding estimations of the mean and SD from the first quartile, and the third quartile can work if the original report provides an interquartile range (IQR) without Q1 and Q3 [7, 10]. Hence, sub-circumstance 3.5 is based on formula 12 in sub-circumstance 3.2. However, formula 11 cannot estimate a mean from IQR without the Q1 and Q3, and the median is regarded as the mean in sub-circumstance 3.5 if data does not violate the assumption of normality.

#### Circumstance-4: pooled SD from two subgroups

On occasion, investigators are intended to combine two subgroups into a single group so SD in the two subgroups should be pooled together. Two well-established formulas, either proposed by Cohen or presented in the Cochrane Handbook [6, 12]. Under the assumption of homogenous variance, investigators can choose the “Cohen” method to combine the SDs (formula 15). If means are available for each subgroup, investigators can choose the formula introduced in the Cochrane Handbook because the equation takes the mean into account, and the pooled SD will be calculated (formula 16).

#### Circumstance 5: SD for the change score

When studies provide the mean and SD of baseline and post-intervention, investigators can easily calculate the mean of change by subtracting the mean of post-intervention from the mean of baseline. However, it would be difficult to obtain the SD of changing scores. Under the assumption of no variation of outcome measurements, reviewers can follow these steps to acquire SDs for the change score.

- Step-1: Seek for studies investigating the same intervention groups using identical outcome measurements.
- Step-2: Make sure the mean and SD of the baseline, post-intervention, and change score are available.
- Step-3: Calculate the correlation coefficient of the experimental group (CORR_E_) and comparator group (CORR_C_) using formulas 17 and 18.
- Step-4: Obtain the overall CORR by averaging the CORR_E_ and CORR_C_.
- Step-5: Impute SD for the change score using SD at both baseline and post with overall CORR using formula 19.

### Between-group circumstances

#### Circumstance 6: available data is SE of difference between two groups

Within-group SD from SE of the difference between two groups is an approximate estimate using the average SD for each group (formula 20). The “within-group SD” refers to the average SD of two intervention groups. Thus, it is an estimated SD for each treatment group with the same value. This method does not seriously bias the result of meta-analysis since a pooled estimate is usually based on mean difference or relative effect and SE of the effect measurement, although formula 20 only produces an approximation of SD.

#### Circumstance 7: only effect estimates with corresponding CI are available

When a difference and its associated CI were reported in a study, SE can be calculated from CI. If investigators would like to convert CI into SE or SD, the calculation firstly has to be based on the statistical test in the primary study. Nevertheless, in the primary report without information on the statistical test, the sample size of each group (≧60 or < 60) might be a clue of the formula selection in terms of the decision between *z* distribution (formula 2) and *t* distribution (formula 4) [6]. Then, SD for each treatment group could be estimated using formula 20.

#### Circumstance 8: only effect estimates with the z score between two groups are available

SE of the difference between the two groups could be estimated by dividing the effect estimate using *z* score (formula 21). Within-group SD in this circumstance can also be estimated using formula 20.

#### Circumstance 9: only effect estimates with the t value between two groups are available

SE of the difference between two groups can be calculated using formula 22. Then, the estimated SD for each treatment group could be obtained using formula 20.

#### Circumstance 10: only effect estimates with *p* value between two groups are available

Investigators have to know what the *p* value for (*z* distribution or *t* distribution) and to estimate within-group SD according to the following steps:

- Step-1: Calculate the *z* score or *t* value from *p* value [13].
- Step-2: Calculate SE of the mean difference between two groups by dividing the effect estimate using *z* score (formula 21) or *t* value (formula 22).
- Step-3: Calculate the average SD for each group based on the SE of mean difference between two groups (formula 21).

## Discussion

Understanding the circumstance and the existing solutions is important to researchers of meta-analysis because appropriate conversion or estimations could increase precision and reduce the risk of bias due to incomplete reporting. On the contrary, imprecision and biased estimates would be due to inappropriate exclusions of some irregular outcome reporting in a meta-analysis [14]. Due to the complicated computations, our team provides a free available spreadsheet calculator for teaching and research based on the formulas and scenarios in the present article. Based on statistics standpoints, we placed the between-group circumstance after the within-group circumstance and order the sequence of sub-circumstances in the Excel tool after the working group meeting. The tool could help investigators without statistical background to estimate or convert data more appropriately. Before using the tool, however, investigators ought to contact corresponding authors to obtain desired statistics. If the incomplete reporting is out of the circumstances in the present article, investigators might use multiple imputations based on a sufficient number of studies with complete information [14]. Then, investigators can only perform qualitative synthesis if they do not receive a response from the original authors and imputation cannot be succeeded due to insufficient studies. To keep the quality of meta-analysis, involving statisticians or experienced researchers in evidence-based practice or study is still recommended [6, 15].

## Supplementary Information

**Additional file 1:** **Supplementary file 1.** Search for relevant systematic reviews. **Supplementary file 2.** List of reviewed textbook and systematic review. **Supplementary file 3.** Resource of the DECoMA file. **Supplementary file 4.** Formulae of data estimation and conversion.

## Data Availability

Data in this study are available to other researchers upon reasonable request to corresponding authors C.F.C. and Y.E.K.

## Abbreviations

CI: Confidence interval
CORR: Correlation coefficient
IQR: Interquartile range
Q1: The first quartile
Q3: The third quartile
SD: Standard deviation
SE: Standard error

## References

[1] Niforatos JD, Weaver M, Johansen ME (2019). Assessment of publication trends of systematic reviews and randomized clinical trials, 1995 to 2017. JAMA Intern Med.

[2] Wallach JD (2019). Meta-analysis metastasis. JAMA Intern Med.

[3] Ioannidis JP (2016). The mass production of redundant, misleading, and conflicted systematic reviews and meta-analyses. Milbank Q.

[4] Chen C-Y, Kang Y-N, Kuo KN, Glasziou P, Chen K-H (2021). Increasing retractions of meta-analyses publications for methodological flaw. Syst Rev.

[5] Chi K-Y, Li M-Y, Chen C, Kang E, Cochrane Taiwan. DECoMA. Taipei: Cochrane Taiwan; 2022.

[6] Higgins JPT TJ, Chandler J, Cumpston M, Li T, Page MJ, Welch VA (editors). Cochrane Handbook for Systematic Reviews of Interventions version 6.3 (updated February 2022). Cochrane, 2022. Available from www.training.cochrane.org/handbook. 2022.

[7] Wan X, Wang W, Liu J, Tong T (2014). Estimating the sample mean and standard deviation from the sample size, median, range and/or interquartile range. BMC Med Res Methodol.

[8] Hozo SP, Djulbegovic B, Hozo I (2005). Estimating the mean and variance from the median, range, and the size of a sample. BMC Med Res Methodol.

[9] Wan X, Wang W, Liu J, Tong T (2014). Estimating the sample mean and standard deviation from the sample size, median, range and/or interquartile range. BMC Med Res Methodol.

[10] Luo D, Wan X, Liu J, Tong T (2018). Optimally estimating the sample mean from the sample size, median, mid-range, and/or mid-quartile range. Stat Methods Med Res.

[11] Bland M (2015). Estimating mean and standard deviation from the sample size, three quartiles, minimum, and maximum. Int J Stat Med Res.

[12] Cohen J (1992). Statistical power analysis. Curr Direct Psychol Sci.

[13] Lin JT (1989). Approximating the normal tail probability and its inverse for use on a pocket calculator. J R Stat Soc Series C.

[14] Weir CJ, Butcher I, Assi V, Lewis SC, Murray GD, Langhorne P (2018). Dealing with missing standard deviation and mean values in meta-analysis of continuous outcomes: a systematic review. BMC Med Res Methodol.

[15] Aromataris E, Munn Z (2020). JBI manual for evidence synthesis.

