# Supplementary material for: Ten circumstances and solutions for finding the sample mean and standard deviation for meta-analysis
Source: Syst Rev. 2023 Apr 1;12:62. doi: 10.1186/s13643-023-02217-1 (PMC10068165; doi:10.1186/s13643-023-02217-1)
Supplement: Supplementary file 1 — Additional file 1: Supplementary file 1. Search for relevant systematic reviews. Supplementary file 2. List of reviewed textbook and systematic review. Supplementary file 3. Resource of the DECoMA file. Supplementary file 4. Formulae of data estimation and conversion. [file 13643_2023_2217_MOESM1_ESM.pdf]

# SUPPLEMENTARY FILE

## Ten circumstances and solutions of finding sample mean and standard deviation for meta-analysis

Running title: Finding sample mean and standard deviation

**Kuan-Yu Chi** M.D. [1], **Man-Yun Li** M.D.[1], **Chiehfeng Chen** M.D., Prof.[2,3,4,5], **Enoch Kang** Consultant[4,5,6,7], **Cochrane Taiwan**

1. School of Medicine, College of Medicine, Taipei Medical University, Taipei, Taiwan
2. Department of Public Health, School of Medicine, College of Medicine, Taipei Medical University, Taipei, Taiwan
3. Division of Plastic Surgery, Department of Surgery, Wan Fang Hospital, Taipei Medical University, Taipei, Taiwan
4. Cochrane Taiwan, Taipei Medical University, Taipei, Taiwan
5. Evidence-Based Medicine Center, Wan Fang Hospital, Taipei Medical University, Taipei, Taiwan
6. Research center of big data and meta-analysis, Wan Fang Hospital, Taipei Medical University, Taipei, Taiwan
7. Institute of Health Policy and Management, College of Public Health, National Taiwan University, Taipei, Taiwan

**Supplementary file 1** Search for relevant systematic reviews.

**Supplementary file 2** List of reviewed textbook and systematic review.

**Supplementary file 3** Resource of the DECoMA file.

**Supplementary file 4** Formulae of data estimation and conversion.

**Supplementary file 1** Search for relevant systematic reviews.

## Primary:

#01 missing[ti]
#02 incomplete[ti]
#03 value*[ti]
#04 data[ti]
#05 mean[ti]
#06 "standard deviation"[ti]
#07 SD[ti]
#08 systematic review
#09 #01 OR #02
#10 #03 OR #04 OR #05 OR #06 OR #07
#11 #09 AND #10 AND #08

## PubMed:

(missing[ti] OR incomplete[ti]) AND (value*[ti] OR data[ti] OR mean[ti] OR "standard deviation"[ti] OR SD[ti]) AND systematic review

**Supplementary file 2** List of reviewed textbook and systematic review.

## Textbook:


1. Aromataris E, Munn Z. JBI manual for evidence synthesis. JBI: Adelaide, Australia. 2020.
2. Borenstein M, Hedges LV, Higgins JP, Rothstein HR. Introduction to meta-analysis. John Wiley & Sons; 2021.
3. Higgins JPT TJ, Chandler J, Cumpston M, Li T, Page MJ, Welch VA (editors). Cochrane Handbook for Systematic Reviews of Interventions version 6.3 (updated February 2022). Cochrane, 2022. Available from www.training.cochrane.org/handbook. 2022.


## Systematic review:


1. Wiebe N, Vandermeer B, Platt RW, Klassen TP, Moher D, Barrowman NJ. A systematic review identifies a lack of standardization in methods for handling missing variance data. J Clin Epidemiol. 2006;59(4):342-53. doi: 10.1016/j.jclinepi.2005.08.017.
2. Alshurafa M, Briel M, Akl EA, Haines T, Moayyedi P, Gentles SJ, et al. Inconsistent definitions for intention-to-treat in relation to missing outcome data: systematic review of the methods literature. PLoS One. 2012;7(11):e49163. doi: 10.1371/journal.pone.0049163.
3. Eekhout I, de Boer RM, Twisk JW, de Vet HC, Heymans MW. Missing data: a systematic review of how they are reported and handled. Epidemiology. 2012;23(5):729-32. doi: 10.1097/EDE.0b013e3182576cdb.
4. Díaz-Ordaz K, Kenward MG, Cohen A, Coleman CL, Eldridge S. Are missing data adequately handled in cluster randomised trials? A systematic review and guidelines. Clin Trials. 2014;11(5):590-600. doi: 10.1177/1740774514537136.
5. Gewandter JS, McDermott MP, McKeown A, Smith SM, Williams MR, Hunsinger M, et al. Reporting of missing data and methods used to accommodate them in recent analgesic clinical trials: ACTTION systematic review and recommendations. Pain. 2014;155(9):1871-7. doi: 10.1016/j.pain.2014.06.018.
6. Li T, Hutfless S, Scharfstein DO, Daniels MJ, Hogan JW, Little RJ, et al. Standards should be applied in the prevention and handling of missing data for patient-centered outcomes research: a systematic review and expert consensus. J Clin Epidemiol. 2014;67(1):15-32. doi: 10.1016/j.jclinepi.2013.08.013.
7. Adewuyi TE, MacLennan G, Cook JA. Non-compliance with randomised allocation and missing outcome data in randomised controlled trials evaluating surgical interventions: a systematic review. BMC Res Notes. 2015;8:403. doi: 10.1186/s13104-015-1364-9.
8. Masconi KL, Matsha TE, Echouffo-Tcheugui JB, Erasmus RT, Kengne AP. Reporting and handling of missing data in predictive research for prevalent undiagnosed type 2 diabetes mellitus: a systematic review. Epma j. 2015;6(1):7. doi: 10.1186/s13167-015-0028-0.
9. Spineli LM, Pandis N, Salanti G. Reporting and handling missing outcome data in mental health: a systematic review of Cochrane systematic reviews and meta-analyses. Res Synth Methods. 2015;6(2):175-87. doi: 10.1002/jrsm.1131.
10. Batson S, Burton H. A Systematic Review of Methods for Handling Missing Variance Data in Meta-Analyses of Interventions in Type 2 Diabetes Mellitus. PLoS One. 2016;11(10):e0164827. doi: 10.1371/journal.pone.0164827.
11. Fiero MH, Huang S, Oren E, Bell ML. Statistical analysis and handling of missing data in cluster randomized trials: a systematic review. Trials. 2016;17:72. doi: 10.1186/s13063-016-1201-z.
12. Hussain JA, White IR, Langan D, Johnson MJ, Currow DC, Torgerson DJ, et al. Missing data in randomized controlled trials testing palliative interventions pose a significant risk of bias and loss of power: a systematic review and meta-analyses. J Clin Epidemiol. 2016;74:57-65. doi: 10.1016/j.jclinepi.2015.12.003.
13. Ibrahim F, Tom BD, Scott DL, Prevost AT. A systematic review of randomised controlled trials in rheumatoid arthritis: the reporting and handling of missing data in composite outcomes. Trials. 2016;17(1):272. doi: 10.1186/s13063-016-1402-5.
14. Mercieca-Bebber R, Palmer MJ, Brundage M, Calvert M, Stockler MR, King MT. Design, implementation and reporting strategies to reduce the instance and impact of missing patient-reported outcome (PRO) data: a systematic review. BMJ Open. 2016;6(6):e010938. doi: 10.1136/bmjopen-2015-010938.

15. Hussain JA, Bland M, Langan D, Johnson MJ, Currow DC, White IR. Quality of missing data reporting and handling in palliative care trials demonstrates that further development of the CONSORT statement is required: a systematic review. J Clin Epidemiol. 2017;88:81-91. doi: 10.1016/j.jclinepi.2017.05.009.
16. Sullivan TR, Yelland LN, Lee KJ, Ryan P, Salter AB. Treatment of missing data in follow-up studies of randomised controlled trials: A systematic review of the literature. Clin Trials. 2017;14(4):387-95. doi: 10.1177/1740774517703319.
17. Malla L, Perera-Salazar R, McFadden E, Ogero M, Stepniewska K, English M. Handling missing data in propensity score estimation in comparative effectiveness evaluations: a systematic review. J Comp Eff Res. 2018;7(3):271-9. doi: 10.2217/cer-2017-0071.
18. Rabe BA, Day S, Fiero MH, Bell ML. Missing data handling in non-inferiority and equivalence trials: A systematic review. Pharm Stat. 2018;17(5):477-88. doi: 10.1002/pst.1867.
19. Shivasabesan G, Mitra B, O'Reilly GM. Missing data in trauma registries: A systematic review. Injury. 2018;49(9):1641-7. doi: 10.1016/j.injury.2018.03.035.
20. Weir CJ, Butcher I, Assi V, Lewis SC, Murray GD, Langhorne P, et al. Dealing with missing standard deviation and mean values in meta-analysis of continuous outcomes: a systematic review. BMC Med Res Methodol. 2018;18(1):25. doi: 10.1186/s12874-018-0483-0.
21. Barretto Dos Santos Lopes Batista K, Thiruvenkatachari B, O'Brien K. Intention-to-treat analysis: Are we managing dropouts and missing data properly in research on orthodontic treatment? A systematic review. Am J Orthod Dentofacial Orthop. 2019;155(1):19-27.e3. doi: 10.1016/j.ajodo.2018.08.013.
22. Richter S, Stevenson S, Newman T, Wilson L, Menon DK, Maas AIR, et al. Handling of Missing Outcome Data in Traumatic Brain Injury Research: A Systematic Review. J Neurotrauma. 2019;36(19):2743-52. doi: 10.1089/neu.2018.6216.
23. Rosenberg M, Townes A, Taylor S, Luetke M, Herbenick D. Quantifying the magnitude and potential influence of missing data in campus sexual assault surveys: A systematic review of surveys, 2010-2016. J Am Coll Health. 2019;67(1):42-50. doi: 10.1080/07448481.2018.1462817.
24. Carroll OU, Morris TP, Keogh RH. How are missing data in covariates handled in observational time-to-event studies in oncology? A systematic review. BMC Med Res Methodol. 2020;20(1):134. doi: 10.1186/s12874-020-01018-7.
25. Mosha NR, Aluko OS, Todd J, Machekano R, Young T. Analytical methods used in estimating the prevalence of HIV/AIDS from demographic and cross-sectional surveys with missing data: a systematic review. BMC Med Res Methodol. 2020;20(1):65. doi: 10.1186/s12874-020-00944-w.
26. Budhiraja P, Kalot MA, Alayli AE, Dimassi A, Kaplan B, Chakkera HA, et al. Reporting and Handling of Missing Participant Data in Systematic Reviews of Kidney Transplant Studies. Transplantation. 2021;105(8):1708-17. doi: 10.1097/tp.0000000000003503.
27. Hunt NB, Gardarsdottir H, Bazelier MT, Klungel OH, Pajouheshnia R. A systematic review of how missing data are handled and reported in multi-database pharmacoepidemiologic studies. Pharmacoepidemiol Drug Saf. 2021;30(7):819-26. doi: 10.1002/pds.5245.
28. Ueland TE, Carreira DS, Martin RL. Substantial Loss to Follow-Up and Missing Data in National Arthroscopy Registries: A Systematic Review. Arthroscopy. 2021;37(2):761-70.e3. doi: 10.1016/j.arthro.2020.08.007.


**Supplementary file 3** Resource of the DECoMA file.

Please refer to the xlsm file via the following link:
from Wan Fang Hospital (https://www.wanfang.gov.tw/p9_medical_detail.aspx?cu=754)
from Cochrane Taiwan (https://taiwan.cochrane.org/resource/decoma)

**Supplementary file 4** Formulae of data estimation and conversion.

$$SD = \sqrt{N} \times SE \quad \text{......} \quad (1)$$

$$SE = \frac{(upper\ limit - lower\ limit)}{2 \times z\text{-}score} \quad \text{......} \quad (2)$$

$$SD = \sqrt{N} \times \frac{(upper\ limit - lower\ limit)}{2 \times z\text{-}value} \quad \text{......} \quad (3)$$

$$SE = \frac{(upper\ limit - lower\ limit)}{2 \times (t\text{-}value)} \quad \text{......} \quad (4)$$

$$SD = \sqrt{N} \times \frac{(upper\ limit - lower\ limit)}{2 \times (t\text{-}value)} \quad \text{......} \quad (5)$$

Abbreviations for the parameters in formula-6 to 14:

$a$ = the minimum value;
$q1$ = the first quartile;
$m$ = the median value;
$q3$ = the third quartile;
$b$ = the maximal value;
$n$ = the sample size;
$\varnothing$ = the percentile of the standard normal distribution;
$\varnothing^{-1}$ = the percentile of the standard normal distribution.

$$Est.mean \begin{cases} \frac{a+2m+b}{4} & if\ n < 25 \\ Median & if\ n > 25 \end{cases} \quad \text{......} \quad (6)$$

$$Est.SD \begin{cases} \sqrt{\frac{(b-a)^2 + \frac{(a-2m+b)^2}{4}}{12}} & if\ n \le 15 \\ \frac{b-a}{4} & if\ 15 < n \le 70 \\ \frac{b-a}{6} & if\ n > 70 \end{cases} \quad \text{......} \quad (7)$$

$$w\ (n) \sim \frac{4}{4+n^{0.75}} \quad \text{......} \quad (8)$$

$$Est.mean \approx \left(\frac{4}{4+n^{0.75}}\right)\frac{a+b}{2} + \left(\frac{n^{0.75}}{4+n^{0.75}}\right)m \quad \text{......} \quad (9)$$

$$Est.SD \approx \frac{b-a}{2\varnothing^{-1}\left(\frac{n-0.375}{n+0.25}\right)} \quad \text{......} \quad (10)$$

$$Est.Mean \approx \left(0.7 + \frac{0.39}{n}\right)\frac{q1+q3}{2} + \left(0.3 - \frac{0.39}{n}\right)m \quad \text{......} \quad (11)$$

$$Est.SD \approx \frac{q3-q1}{2\emptyset^{-1}(\frac{0.75n-0.125}{n+0.25})} \quad \text{......} (12)$$

$$Est.Mean \approx \left(\frac{2.2}{2.2+n^{0.75}}\right)\frac{a+b}{2} + \left(0.7 - \frac{0.72}{n^{0.55}}\right)\frac{q1+q3}{2} + \left(0.3 + \frac{0.72}{n^{0.55}} - \frac{2.2}{2.2+n^{0.75}}\right)m \quad \text{...} (13)$$

$$Est.SD \approx \frac{b-a}{4\emptyset^{-1}(\frac{n-0.375}{n+0.25})} + \frac{q3-q1}{4\emptyset^{-1}(\frac{0.75n-0.125}{n+0.25})} \quad \text{......} (14)$$

$$SD_{pooled} = \sqrt{\frac{(n_1-1)SD_1^2+(n_2-1)SD_2^2}{n_1+n_2-2}} \quad \text{......} (15)$$

$$SD_{pooled} = \sqrt{\frac{(n_1-1)SD_1^2+(n_2-1)SD_2^2+\frac{n1\times n2}{n1+n2}(mean_1^2+mean_2^2-2\times mean_1\times mean_2)}{n_1+n_2-1}} \quad \text{......} (16)$$

$$CORR_E = \frac{SD_{E,baseline}^2+SD_{E,post}^2-SD_{E,change}^2}{2\times SD_{E,baseline}\times SD_{E,post}} \quad \text{......} (17)$$

$$CORR_C = \frac{SD_{C,baseline}^2+SD_{C,post}^2-SD_{C,change}^2}{2\times SD_{C,baseline}\times SD_{C,post}} \quad \text{......} (18)$$

$$SD_{change} = \sqrt{SD_{baseline}^2 + SD_{post}^2 - 2 \times CORR \times SD_{baseline} \times SD_{Epost}} \quad \text{...} (19)$$

$$SD_{average} = \frac{SE}{\sqrt{\frac{1}{n1}+\frac{1}{n2}}} \quad \text{......} (20)$$

$$SE = \frac{Effect\ estimate}{z-score} \quad \text{......} (21)$$

$$SE = \left|\frac{mean\ difference}{t-value}\right| \quad \text{......} (22)$$
